# Diagnostic Accuracy of Shotgun Metagenomics for Bloodstream Infections Is Influenced by Bioinformatics Workflow Selection

**DOI:** 10.1002/mbo3.70158

**Published:** 2025-12-15

**Authors:** Yajing Song, Christian Kjellander, William Robinson, Lars Öhrmalm, Christian Giske, Peter Gyarmati

**Affiliations:** ^1^ University of South Carolina School of Medicine Greenville, Greenville South Carolina USA; ^2^ Department of Laboratory Medicine Karolinska Institutet Stockholm Sweden; ^3^ Department of Internal Medicine Södersjukhuset Stockholm Sweden; ^4^ PRIMA Barn‐ och Vuxenpsykiatri Stockholm Sweden

## Abstract

Bloodstream infection (BSI) is a severe and often fatal condition, and a major cause of mortality in patients with hematological malignancies due to underlying conditions and anticancer therapy‐induced immunodeficiency. Rapid identification of the causative pathogens is essential as BSI results in worsened prognosis, extended hospitalization, delays or dose reductions in therapy, and may progress to sepsis and septic shock if untreated. Shotgun metagenomics is a culture‐independent technique capable of detecting a wide range of fungal, viral, and bacterial organisms along with their antimicrobial resistance genes. Several studies showed that shotgun metagenomics enables the diagnosis of BSI, specifically in cases where conventional methods/culture‐dependent techniques fail to identify the causative pathogens. However, evaluation of the accuracy of the applied bioinformatics pipelines remains incomplete. This study aimed to compare and optimize four commonly used bioinformatics pipelines (BLAST, Kraken, Metaphlan, RTG Core) for shotgun metagenomics by assessing their accuracy in identifying pathogens in blood samples from patients with hematological malignancies and suspected BSI, with blood culture serving as the reference standard. Our work shows that the selection of bioinformatics pipelines for diagnosing BSI strongly affects the precision of the findings, and an optimized BLAST pipeline was superior to the alternatives, as it was the only method that accurately identified the causative pathogens.

## Graphical abstract

Our work shows that the selection of bioinformatics pipelines for diagnosing bloodstream infection strongly affects the precision of the findings, and an optimized BLAST pipeline was superior to the alternatives, as it was the only method that accurately identified the causative pathogens.

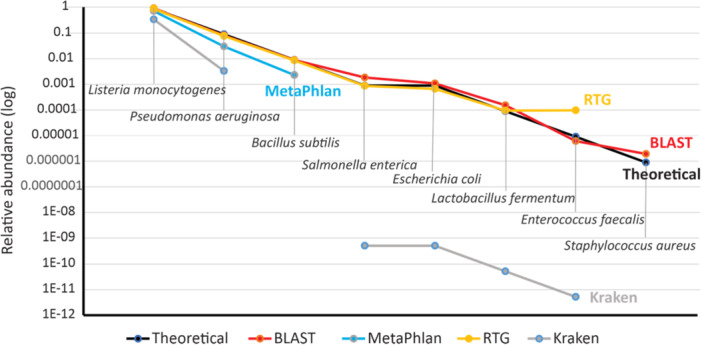

## 1 Introduction

Bloodstream infection (BSI) is a major cause of mortality in patients with hematological malignancies due to the underlying immunological defects and therapy‐induced immunosuppression (de Naurois et al. 2010). BSI results in worsened prognosis, extended hospital stay and increased healthcare costs (Garcia‐Vidal et al. 2018; Dinan et al. 2015). Rapid diagnosis of BSI is essential as patient prognosis improves with early detection (Kalich et al. 2015), while organ dysfunction and septic shock can develop if the infection is not treated in time (Laupland 2013). Blood culture is the reference method to diagnose BSI. However, this assay has a turnaround time of 4‐72 h and therefore it may not be suitable in assisting primary diagnosis and selection of antimicrobials in case of fastidious organisms. In addition, blood culture cannot detect viral, and most fungal pathogens, and microbial interference may hinder polymicrobial detections. These are likely some of the reasons why 70%–90% of blood cultures from febrile neutropenia and 50% of blood cultures from septic shock are negative and the causative agents(s) remain unknown (Hämäläinen et al. 2008; Liesenfeld et al. 2014).

Molecular methods such as PlexID, MagicPlex, SeptiFast, VYOO have been available to detect BSI, but they only detect a subset of pathogens using pre‐designed primers and probes. These amplification‐based molecular techniques are prone to false positivity in BSI diagnosis due to a large amount of human DNA background in blood samples, and false negativity due to mutations or variants not covered by primers and probes (Sinha et al. 2018; Opota et al. 2015; Ecker et al. 2010).

Standard treatment of BSI involves treatment with empirical broad‐spectrum antibiotics, which are inefficient against fungal or viral pathogens, or against antimicrobial‐resistant (AMR) bacteria. As a result, inappropriate antimicrobial use can contribute to an increase of AMR strains. It has been shown that 46% of the empirical antibiotic treatments were inappropriate and may have contributed to a 35% increase in overall mortality when treating BSI (Ammerlaan et al. 2013; Paul et al. 2010). Therefore, accurate characterization of the causative agents could improve the specificity and efficacy of antimicrobial treatments. Using culture‐independent, untargeted techniques such as shotgun metagenomics, it has been shown that BSI in hematological malignancies is rarely monomicrobial, and caused not only by bacteria, but also by fungal and viral pathogens (Gyarmati et al. 2015, 2016; Fida et al. 2021; Ohrmalm et al. 2012). A step towards clinical validation of the method includes an optimization of the bioinformatics pipeline. Metagenomics analysis of blood samples in BSI differs from other specimen types, as blood contains a small amount of microbial DNA, but a large amount of human DNA, which can lead to false‐positive detections (Gyarmati et al. 2015, 2016; Fida et al. 2021). Therefore, in this study we compared the accuracy of 4 shotgun metagenomics software to the gold standard in their accuracy of identifying the causative pathogens in blood samples from patients with hematological malignancies and suspected BSI.

## 2 Methods

### 2.1 Sample Collection and Processing

Blood samples were taken from patients with hematological malignancies and suspected BSI and processed for blood culture as described before (Gyarmati et al. 2016). BSI was defined as an infection manifested by the presence of bacteria in at least one blood culture bottle, or at least two blood culture bottles with the same microorganism growing in the case of common skin contaminants. Commercial BacT‐Alert 3D system with 2–2 aerobic and anaerobic bottles was used (bioMérieux, Marcy l'Etoile, France) for culture. Two‐hundred microliters of the blood samples were processed for DNA extraction and shotgun metagenomics sequencing using the MolYsis Complete5 kit (Molzym Life Science, Bremen, Germany). Sequencing reads were processed for quality control as described before (Gyarmati et al. 2016; Song et al. 2022). Briefly, 1 μg of the eluted DNA was processed to NebNext microbiome enrichment (New England Biolabs, Ipswich, MA, USA) following the manufacturer's instructions. Ten nanograms of DNA was subjected to multiplex displacement amplification, using the GenomiPhi V2 DNA amplification kit (GE Healthcare, Little Chalfont, United Kingdom), with 90 min amplification resulting in 2–4 μg DNA. Two micrograms DNA was used for library preparation with the Nextera XT kit and libraries were processed to a 2*100 base pair PE sequencing on a HiSeq. 2500 instrument. Reads shorter than 30 bp and with Phred quality scores below 30 were discarded using the Fastx toolkit. Paired end reads were merged using the Flash software, while unpaired reads were discarded, with an average read count of 33.5 M/sample (Gyarmati et al. 2016).

### 2.2 Data Analysis

Only sequencing data from blood samples with positive blood cultures were used for the current analysis. Fastq files for BLAST (BLASTn, 18), Kraken (Wood and Salzberg 2014) and Metaphlan (Beghini et al. 2021) were used via the Galaxy platform (Abueg et al. 2024), and all software, including RTG Core v3.12 (Cleary et al. 2014) used default settings, with the exception that the required minimum sequence similarity was set to 90% for all software where it was not the default value. All software were used with their respective native database. BLAST was used with the nt nucleotide database. Microfiltered sterile human blood (“negative blood”, from Sigma‐Aldrich, St. Louis, MO, USA) and no template control (NTC, sterile water) were used as negative controls, and microbial detection was considered valid only if it was not detected in either negative blood or in NTC. The ZymoBIOMICS Microbial Community Standard II (Zymo Research, Irvine, CA, USA) was used as a standardized positive control to evaluate assay performance.

Eta‐squared *(η* ^2^) was used as a statistical measure to quantify the effect size in ANOVA as proportion of total variance (*η* ^2^ = Sum of squares between groups/Total sum of squares), where 0 = none of the variance is explained by the group differences, 1 = all the variance is explained by the group differences (Cohen 1988).

## 3 Results

First, we evaluated the accuracies of different software using a mock microbial community standard consisting of gram‐positive and gram‐negative organisms and yeasts, representing clinically relevant microbes with different cell wall compositions (Zymo Microbial Community Standard (Figure S1). Only BLAST could identify pathogens to the lowest relative abundance (8.9E‐07, *Staphylococcus aureus*), while RTG Core showed a strong correlation down to 8.9E‐05 of relative abundance in comparison to the theoretical distribution (Table 1).

**Table 1:** Relative abundances of the standard microbial control as analyzed by different software, in comparison with the theoretical distribution.

	Theoretical	Blast	Metaphlan	RTG	Kraken
*Listeria monocytogenes*	8.90E‐1	8.97E‐1	7.23E‐1	8.64E‐1	3.40E‐1
*Pseudomonas aeruginosa*	8.90E‐2	8.03E‐2	3.00E‐2	7.65E‐2	3.30E‐3
*Bacillus subtilis*	8.90E‐3	8.82E‐3	2.30E‐3	8.50E‐3	0
*Salmonella enterica*	8.90E‐4	1.84E‐4	0	8.65E‐4	5.17E‐10
*Escherichia coli*	8.90E‐4	1.07E‐3	0	6.54E‐4	5.17E‐10
*Lactobacillus fermentum*	8.90E‐5	1.53E‐5	0	9.36E‐5	5.17E‐11
*Enterococcus faecalis*	8.90E‐6	6.07E‐6	0	9.68E‐5	5.17E‐12
*Staphylococcus aureus*	8.90E‐7	1.90E‐6	0	0	0
*η* ^2^	*	4.25E‐9	3.00E‐3	7.09E‐5	3.20E‐2
*p*‐value	*	> 0.999	0.840	0.975	0.504

*not applicable.

*Note: η* ^2^ and *p*‐value of ANOVA are shown.

Next, we investigated the accuracy of detection by different software in blood samples. Five blood samples were culture positive as described previously (Gyarmati et al. 2016), and all positive blood cultures showed α‐hemolytic Streptococci. As expected, relative abundances for positive detections were low compared to other sample types with a native microbiome (feces, oral swab, etc.), since human genomic background greatly outweighs microbial reads in blood samples in BSI (Gyarmati et al. 2016; Song et al. 2014). Only BLAST was able to accurately detect viridans group streptococci in all blood samples from shotgun metagenomics data that were positive for these organisms on blood culture (Table 2). Even though BLAST was the only software that correctly identified all culture‐positive samples, it also detected the negative blood and NTC samples as false positives.

**Table 2:** Relative abundances of viridans group Streptococci as detected by different software in blood samples.

	Blast	Kraken	RTG	Metaphlan
Sample 1	1.21E‐1	1.74E‐5	0	0
Sample 2	2.87E‐2	0	0	0
Sample 3	6.49E‐1	2.03E‐2	2.74E‐1	2.30E‐2
Sample 4	4.39E‐2	1.69E‐5	0	0
Sample 5	2.39E‐2	0	0	0
Negative blood	1.03E‐1	0	0	0
No template control	2.59E‐1	0	0	0

*Note:* Green indicates the highest, while red indicates the lowest abundances.

### 3.1 Reduction of False‐Positive Signals

We then investigated two different approaches to reduce or eliminate false positive signals from BLAST results. First, we tested whether the removal of human sequences improves detection due to less background noise. Sequences were mapped to the human genome (build Hg_19) before mapping reads to the nt database with e‐value set to 10E‐6. There were no significant differences between the relative abundances of viridans group streptococci detections (*p* = 0.15) with or without the prior removal of human sequences.

**Figure 1:**
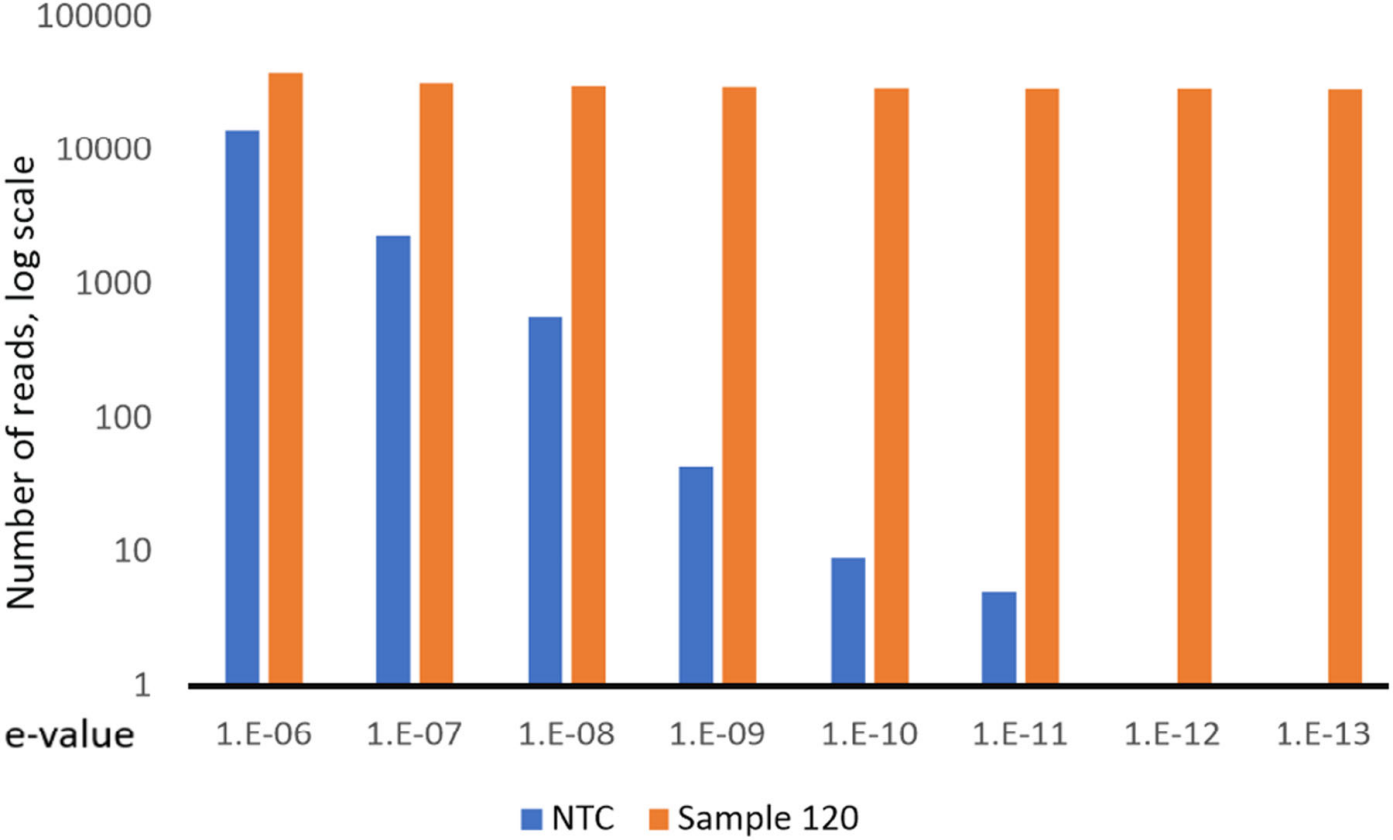
Number of reads specific to viridans group streptococci by different e‐values (from 10 to 6 to 10–13) have been tested using no template control (blue columns, NTC) and sample 3 (red columns).

Second, we tested whether lowering the e‐value improves detection specificity. E‐value (Expect value) is a parameter in BLAST describing how significant the match is, and lowering the e‐value leads to more stringent search criteria. Accuracy is critical for pathogen detection in blood samples, as human reads can outnumber microbial reads to 1:1 M or higher (Song et al. 2014), which can lead to false‐positive detections. Lowering the e‐value helped filter out false positives from no template control without affecting the accuracy of the method (Figure 1). At e‐value level 10E‐12, there was a single read assigned to NTC. All culture‐positive samples were positive and had the highest abundance for viridans streptococci at e‐value level 10E‐13, while NTC and negative blood samples were negative with 0 assigned read. Other microbial detections were in lower abundances and were not clinically relevant (e.g., *Alicycliphilus denitrificans, Tetragenococcus halophilus*).

## 4 Discussion

Blood culture is the current reference standard for the detection of bloodstream infections, and therefore is considered as the most reliable and accurate method overall. Reference standards are not without shortcomings, as blood culture takes longer time to perform than molecular methods, and turnaround time can be up to 3 days for slow‐growing/fastidious microorganisms. Using blood culture also presumes that all causative pathogens can grow in the medium, while many pathogenic microorganisms have specialized growth requirements (Stewart 2012). In addition, blood culture can result in monomicrobial detection due to microbial interference (Lagier et al. 2015), despite many studies showing that BSI is often polymicrobial (Gyarmati et al. 2015, 2016; Fida et al. 2021; Ohrmalm et al. 2012). These drawbacks may contribute to the low positivity rate of blood cultures, as only 10‐30% of blood cultures are positive in sepsis. Even in patients with septic shock, the causative agent remains unknown due to negative blood culture results in up to 50% of the cases (Li et al. 2021; Gupta et al. 2016).

Shotgun metagenomics emerged as a novel technology with the potential to address the challenges related to BSI detection. As a culture‐independent method, it can detect a wide variety of pathogens, and their antimicrobial profiles with great precision, with a turnaround time of 4–40 h. However, blood as a sample poses special challenges to shotgun metagenomics, as it contains a large amount of host DNA and a small amount of microbial DNA, making this method less effective for BSI detection because most of the sequencing power is directed toward background signals (Gyarmati et al. 2016). Metagenomic analysis of blood samples also poses unique obstacles for bioinformatics methods, as they have been optimized for samples where microbes are present in significant proportion, such as fecal matter, bronchoalveolar fluid, and oral and skin swabs, among others. In an effort toward standardization and clinical validation of shotgun metagenomics, an optimized bioinformatics pipeline is required. We compared four bioinformatics pipelines commonly used for analyzing shotgun metagenomics data.

We only included culture‐positive samples, as the aim of this study was to use culture data as benchmarks. Although all pipelines included are widely used in metagenomic analyses, they rely on distinct bioinformatic principles. Kraken classifies reads by mapping exact k‐mers to a reference database and assigning taxonomy via the lowest common ancestor (LCA) algorithm; MetaPhlAn estimates community composition from clade‐specific marker genes; BLAST uses a heuristic alignment approach; and RTG Core applies a dynamic‐programming edit‐distance model (Cock et al. 2015; Wood and Salzberg 2014; Beghini et al. 2021; Cleary et al. 2014). These methodological differences may explain the variation in performance observed across control and test samples. BLAST requires the largest hardware and database (Salzberg and Wood 2021) of the four pipelines included in the study. It was the only one that reached 100% concordance with blood culture results, but required several optimization steps to avoid false‐positive signals. These results will guide the integration of shotgun metagenomics into clinical practice, and will allow an improved detection of BSI for rapid antimicrobial treatment as part of precision medicine.

This study has several limitations. We used blood culture results as a reference since this method is the current gold standard, even though its low positivity rate and limited polymicrobial detection are well known. Twenty‐two blood samples were subjected to shotgun metagenomics in the original study (Gyarmati et al. 2016), which contained 5 samples positive for blood culture. The low positivity rate of blood cultures highlights the importance of complementary diagnostic techniques in the diagnosis of BSI (Hämäläinen et al. 2008; Liesenfeld et al. 2014). The BLAST software resulted in a 95% confidence interval of 0.61‐1 using the Wilson score interval (Agresti et al. 1998) by correctly identifying pathogens in blood culture positive samples, along with positive and negative controls. All blood cultures included in this study were positive for viridans group streptococci; however, streptococci are common causes of BSI. In addition, this study tested the software and its respective native database, but did not combine them due to computational or software limitations.

## 5 Conclusion

The choice of metagenomics pipeline for analyzing shotgun metagenomics data for BSI samples strongly influences the result. In this study, an optimized BLAST pipeline with stringent e‐value was shown to be superior to the other tested options and provided complete concordance with the gold standard without false negative or false positive detection.

## Author Contributions

**Yajing Song:** conceptualization (supporting), writing – review and editing (supporting), formal analysis (supporting). **Christian Kjellander:** data curation (lead), writing – review and editing (supporting), formal analysis (supporting). **William Robinson:** writing – review and editing (supporting), formal analysis (supporting). **Lars Öhrmalm:** data curation (supporting), writing – review and editing (supporting), formal analysis (supporting). **Christian Giske:** conceptualization (supporting), writing – review and editing (supporting), supervision (supporting). **Peter Gyarmati:** conceptualization (lead), writing – review and editing (lead), writing – original draft (lead), supervision (lead).

## Ethics Statement

All patients provided written, informed consent. The study and all experimental protocols used in this study was approved by The Regional Ethical Review Board, Stockholm (2012/1929‐31/1) and were carried out in accordance with the approved guidelines.

## Conflicts of Interest

The authors declare no conflicts of interest.

## Supporting information


**Supplementary Figure 1.** Relative abundances of the microbial community standard as determined by different software, compared to the theoretical distribution (log scale).

## Data Availability

Sequencing data has been uploaded to NCBI Sequencing Reads Archives under accession number PRJNA1268383.
