# Supplementary material for: Diagnostic Accuracy of Shotgun Metagenomics for Bloodstream Infections Is Influenced by Bioinformatics Workflow Selection
Source: Microbiologyopen. 2025 Dec 15;14(6):e70158. doi: 10.1002/mbo3.70158 (PMC12705909; doi:10.1002/mbo3.70158)
Supplement: Supplementary file 1 — Supplementary Figure 1. Relative abundances of the microbial community standard as determined by different software, compared to the theoretical distribution (log scale). [file MBO3-14-e70158-s001.docx]

SUPPLEMENTARY MATERIAL


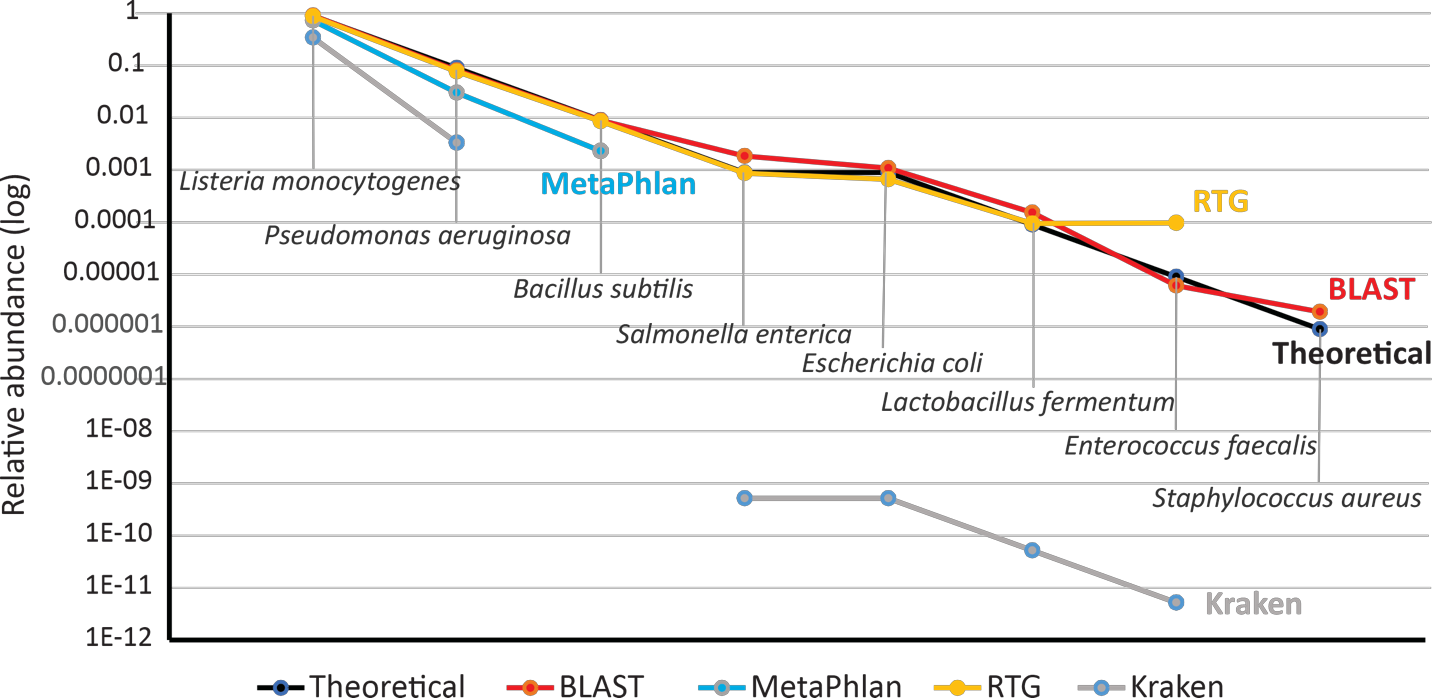


Supplementary Figure 1. Relative abundances of the microbial community standard as determined by different software, compared to the theoretical distribution (log scale).
